# Transmission of Pandemic Influenza A (H1N1) Virus in a Train in China

**DOI:** 10.2188/jea.JE20100119

**Published:** 2011-07-05

**Authors:** Fuqiang Cui, Huiming Luo, Lei Zhou, Dapeng Yin, Canjun Zheng, Dingming Wang, Jian Gong, Gang Fang, Jianfeng He, Jeffrey McFarland, Hongjie Yu

**Affiliations:** 1 Chinese Center for Disease Control and Prevention, Beijing, China; 2 Guizhou Center for Disease Control and Prevention, Guiyang, China; 3 Guangxi Center for Disease Control and Prevention, Nanning, China; 4 Sichuan Center for Disease Control and Prevention, Chengdu, China; 5 Guangdong Center for Disease Control and Prevention, Guangzhou, China; 6 China-U.S. Collaborative Program on Emerging and Re-emerging Infectious Diseases, Beijing, China; 7 Influenza Division, National Center for Immunization and Respiratory Diseases, Centers for Disease Control and Prevention, Atlanta, USA

**Keywords:** pandemic influenza A (H1N1), outbreak, train

## Abstract

**Background:**

Pandemic influenza A (H1N1) virus emerged in North America in April 2009 and spread globally. We describe the epidemiology and public health response to the first known outbreak of 2009 H1N1 in a train, which occurred in June 2009 in China.

**Methods:**

After 2 provinces provided initial reports of 2009 H1N1 infection in 2 persons who had travelled on the same train, we conducted a retrospective epidemiologic investigation to collect information from the passengers, crew members, contacts, and health care providers. We explored the source of infection and possible routes of transmission in the train. All cases were confirmed by real-time reverse transcription polymerase chain reaction testing.

**Results:**

Train #1223 traveled 40 hours, made 28 stops in 4 Chinese provinces, and boarded 2555 passengers, who logged a total of 59 144 person-hours of travel time. Nineteen confirmed 2009 H1N1 cases were identified. Of these, 13 were infected and developed symptoms on the train and 6 occurred among contacts who developed illness during medical monitoring. In addition, 3 asymptomatic cases were identified based on RT-PCR testing of respiratory swabs from contacts. The attack rate among contacts of confirmed cases in the same car was higher than that among contacts in other cars (3.15% vs. 0%, *P* < 0.001). Attack rates increased with exposure time.

**Conclusions:**

Close contact and long exposure may have contributed to the transmission of 2009 H1N1 virus in the train. Trains may have played an important role in the 2009 influenza pandemic.

## INTRODUCTION

Every year, people take more than 2.3 billion train trips in China, creating a temporary community and the opportunity for the spread of communicable diseases in trains. The large temporary community created during the annual Hajj, when millions of Muslim pilgrims from all over the world travel by airplane, ship, train, bus, and cars to Mecca has also experienced outbreaks,^1^ including cholera, plague, smallpox, meningococcal meningitis, and infectious diarrheas.^2^^,^^3^ Transmission of respiratory infectious diseases on airplanes and ships has been reported^4^^,^^5^; however, reports of such transmission on trains are less common.^6^

Pandemic 2009 influenza A (H1N1) virus (hereafter referred to as 2009 H1N1 virus) spread rapidly and resulted in millions of laboratory-confirmed cases. More than 18 000 deaths were reported from more than 200 countries. The global distribution of the pandemic prompted the World Health Organization (WHO) to declare the first influenza pandemic of the 21st century in June 2009. Influenza transmission is thought to occur primarily via droplet spread and contact transmission, although droplet nuclei transmission is also likely.^7^^–^^9^ Transmission of 2009 H1N1 and other respiratory viruses is facilitated by crowded and confined environments.^10^^–^^12^ The first confirmed case of 2009 H1N1 infection was identified on 11 May 2009 in mainland China.^13^^,^^14^ Since then, transmission patterns evolved rapidly from a few imported cases to sustained community level transmission and outbreaks.^15^ Through 21 July 2009, a total of 1762 confirmed 2009 H1N1 cases were reported in the country, 75% of which were imported cases.

On 10 June 2009, the Hainan Provincial Center for Disease Control (CDC) reported to the China CDC a confirmed index case of 2009 H1N1 infection in a 20-year-old woman. Initial case investigation indicated that she had left Chengdu City, Sichuan Province by train 1223 on the afternoon of 7 June and arrived in Guangzhou City, Guangdong Province on the morning of 9 June. On 10 June, the investigation of another confirmed 2009 H1N1 case by the Guangzhou provincial CDC indicated that this person had also traveled in the same car of the same train with the index case. China CDC immediately launched an epidemiologic investigation to identify more cases and characterize transmission in the train. This report describes the epidemiologic findings and public health response to the first known outbreak of 2009 H1N1 in a train in China.

## METHODS

### Surveillance and investigation

Case definitions: Acute respiratory illness (ARI) was defined as a recent onset of fever (temperature ≥37.5°C) or at least 1 of the following symptoms: rhinorrhea, nasal congestion, sore throat, or cough. A suspected case of 2009 H1N1 was defined as a person with ARI and 1 of the following: illness onset within 7 days of travel to an area with confirmed cases or within 7 days of close contact with a confirmed case. A confirmed case was defined as a patient with ARI and laboratory evidence of 2009 H1N1 infection diagnosed by real-time reverse transcription polymerase chain reaction (rRT-PCR) testing of respiratory specimens.^15^

Routine surveillance and reporting of suspected cases are done through the National Notifiable Disease Reporting System, as stipulated by law, beginning on 30 April 2009.^14^ In addition, to increase case finding related to the train outbreak, we delivered public announcements in the 4 provinces that were on the route of the train. The announcements requested passengers and staff to present themselves to local public health authorities for evaluation. Professional staff at local CDCs administered a screening questionnaire to respondents. Respondents who had a history of ARI or who had been in the train car with a confirmed case-patient were investigated. Trained interviewers collected information from passengers, crew, and health care workers by using a structured questionnaire that included items on demographic characteristics, symptoms, date of illness onset, and travel history. Quarantine either at designated hospitals or at home was imposed when professionals confirmed the presence of symptoms. We also collected information from the Chinese Ministry of Railways to investigate the internal environment of the train, including air conditioning, the cafeteria, seating locations, capacity, timetable, crew, passengers, and structure.

### Laboratory testing

Following a standard protocol, respiratory specimens (nasal, throat, and nasopharyngeal swabs) were collected from suspected case-patients and placed in sterile viral transport media for 2009 H1N1 testing.^16^ RNA was extracted from specimens using the RNeasy Mini Kit (Qiagen, Valencia, CA, USA) per the manufacturer’s protocol and tested by rRT-PCR following the US CDC protocol.^17^ Assays were performed in provincial Centers for Disease Control and Prevention and confirmed by the National Influenza Center of the China CDC.

### Medical observation of close contacts

A close contact was defined as a person known to have been within 2 meters of a confirmed 2009 H1N1 case-patient for any length of time during the case’s infectious period. This included household and social contacts and health care workers who were assessed to have used suboptimal personal protective equipment. In the absence of viral shedding data, the infectious period for a confirmed case-patient was defined as the time period from 1 day before illness to either 7 days after illness onset or resolution of symptoms, whichever was longer.

### Statistical analysis

Attack rate was defined as the proportion of passengers investigated who developed a syndrome compatible with a confirmed case. Relative risk (RR) was defined as the risk of ARI among persons exposed to a confirmed case or exposure time; 95% confidence intervals were used to describe the range of attack rates or RR. The chi-square (χ^2^) test was used for testing statistical significance. A *P* value less than 0.05 was regarded as statistically significant.

## RESULTS

### The train

Train 1223 departed from Chengdu City, Sichuan Province at 17:06 on 7 June 2009, stopped in Guizhou Province, then Guangxi Province, and finally arrived in Guangzhou City, Guangdong province at 09:09 on 9 June 2009, after 28 stops on its 2445-kilometer journey—a travel time of 40 hours and 31 minutes. The train had 18 cars: car 1 was a secure car for engineers only, cars 2 through 9 had third-class seats, car 10 was a dining car, car 11 had first-class sleeping beds, car 12 and cars 14 through 17 had second-class sleeping beds, car 18 was for crew use only, and car 19 was an isolation car for suspected 2009 H1N1 case-patients (this was not used during the present journey as all 2009 H1N1 case-patients were diagnosed later). The train did not have a car 13. Each sleeping car had 10 rooms, with 4 beds per room in first class and 6 beds per room in second class.

The full capacity of the train was 1328 passengers, including 116 seats for each third-class car, 60 beds for each second-class car, and 40 beds for the first-class car. There were 52 employees on the train, including 2 for the first-class car, 2 for each second-class car, and 2 for each third-class car. All service personnel worked 8-hour shifts alternating with 8-hour breaks and walked through their car at least once every 30 minutes. The windows remained closed in first class (car 11) and the crew car (car 18) because those cars were air-conditioned. The windows could be opened in other cars (2–9, 10, 12, and 14–17). Smoking was prohibited in cars 11 through 17. The initial station, in Chengdu City, is a very crowded station in which more than 50 000 passengers transit each day.

### Demographic characteristic of cases

**Figure 1.**
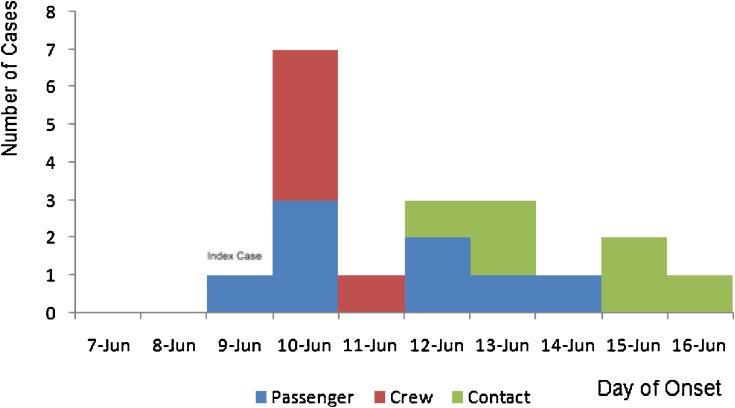
Number of confirmed cases of 2009 H1N1 infection in train 1223 by date of onset, China 2009

From 9–16 June 2009, a total of 19 confirmed cases were identified through surveillance, including 8 passengers and 5 crew in the train, and 6 non-train contacts after journey. Among the 19 cases, 16 were identified from 28 stops (Table 1), including 13 case-patients reported by local hospitals during their consultation and 3 who presented themselves to local CDCs; the remaining 3 of the 19 were later confirmed during medical isolation. Additionally, 3 asymptomatic cases were identified during medical observation. Confirmed cases were detected in 4 provinces, namely, Sichuan (3), Guizhou (2), Guangxi (4), and Guangdong (10), and peak onset was on 10 June (Figure 1). Of the 19 confirmed cases, 11 were female (58%). Age ranged from 17 to 49 years (mean: 27). Six (32%) were students, 3 (16%) public service workers, 3 (16%) civil servants, 2 (11%) in business services, 1 (5%) nurse, and 4 (20%) in other professions.

**Table 1.** Characteristics of confirmed 2009 H1N1 cases associated with train 1223, China, 2009

Case No.	Sex/Age	Time of onset	Relationship	Car No.	Destination
1	Female/20	6-9-09 11:00 AM	Passenger	12	Guangzhou
2	Male/21	6-10-09 3:00 AM	Passenger	12	Guangzhou
3	Female/36	6-10-09 5:00 AM	Crew	12	Liupanshui
4	Female/22	6-10-09 11:00 AM	Crew	12	Liupanshui
5	Female/21	6-10-09 11:30 AM	Passenger	12	Guangzhou
6	Male/49	6-10-09 11:30 AM	Passenger	12	Guangzhou
7	Female/23	6-10-09 4:00 PM	Crew	11	Chengdu
8	Female/27	6-10-09 9:00 PM	Crew	Supervisor	Chengdu
9	Female/23	6-11-09 12:00 PM	Crew	11	Chengdu
10	Female/25	6-12-09 12:00 PM	Passenger	14	Liuzhou
11	Male/40	6-12-09 12:00 PM	Passenger	11	Dujun
12	Male/22	6-12-09 1:00 AM	Contact of Case 2	—	Guangzhou
13	Male/18	6-13-09 12:00 PM	Contact of Case 5	—	—
14	Female/34	6-13-09 12:00 PM	Passenger	11	Dujun
15	Female/29	6-13-09 10:00 AM	Contact of Case 2	—	—
16	Male/38	6-14-09 12:00 PM	Passenger	14	Liuzhou
17	Male/17	6-15-09 12:00 PM	Contact of Case 13	—	—
18	Male/18	6-15-09 12:00 PM	Contact of Case 13	—	—
19	Female/36	6-16-09 12:00 PM	Contact of Case 15	—	—
20^a^	Male/18	6-17-09 17:00 PM	Contact of Case 5	—	—
21^a^	Male/18	6-17-09 17:00 PM	Contact of Case 13	—	—
22^a^	Male/51	6-17-09 17:00 PM	Contact of Case 13	—	—

^a^Asymptomatic case.

### Timeline of the cases

The first (index) case was a 20-year-old woman who boarded at Chengdu City (first station) at 17:07 on 7 June in car 12 and got off at Guangzhou City (last station) at 9:09 on 9 June. She traveled 2445 kilometers through 28 stops during a period of 40 hours and 31 minutes. Among the 19 cases, 7 cases reported a clear, unique exposure date. Of these, the median incubation period was estimated at 3 days (range: 0–3). Sixteen of the 19 case-patients presented themselves at the hospital on the day of appearance of symptoms (range in duration from onset to presentation for treatment: 0 to 4 days). Of the 19 cases identified, the estimated median interval was 2 days from the first to the second generation (range: 1–4), the second to the third generation (range: 1–3), and the third to the fourth generation (range: 2–3).

### Distance, model, and time of transmission

**Figure 2.**
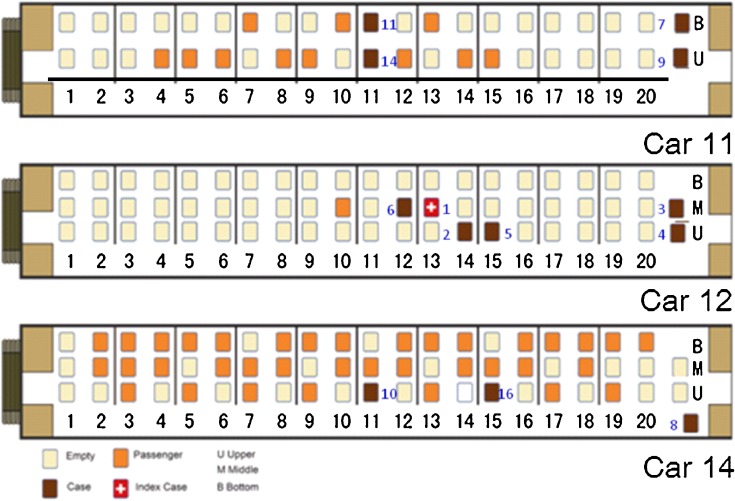
Distribution of confirmed 2009 H1N1 cases in train 1223, China, 2009. Case numbering is shown in blue.

**Figure 3.**
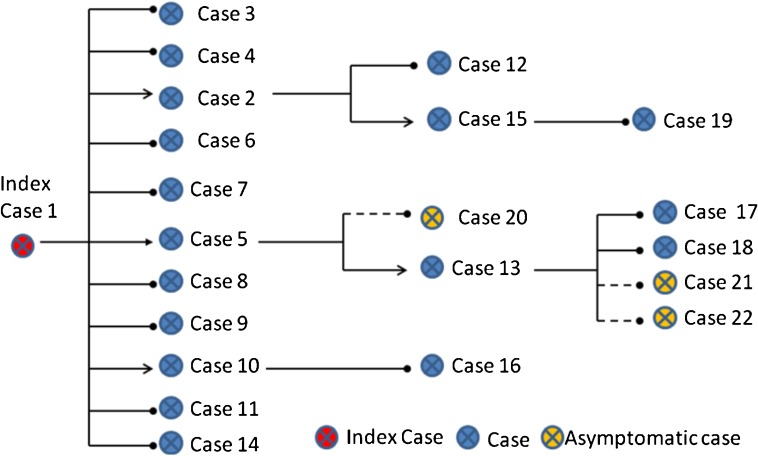
Transmission model of cases

Among the 19 case-patients, 13 (68%) took train 1223. Of these, 2 were seated face-to-face in car 11, 4 had seats clustered in the same car (car 12), 2 were in car 14, and 5 were crew members attending to these passengers (2 in car 11, 2 in car 12, and 1 was head of crew, Figure 2). All confirmed case-patients reported an exposure in car 11, car 12, or car 14. None reported any other exposure to a confirmed case or ARI patient before boarding the train. We ultimately identified 22 infections induced by train exposures; the transmission model and the most likely links between reported cases are shown in Figure 3.

Of the 2555 passengers who boarded the train, 1759 left the train at 1 of the 28 stops; 796 passengers remained on board until the end of the journey. Of all passengers, 685 (26.8%) were investigated. The attack rate increased with time and distance travelled, from 0.56% for the shortest exposure time (13 hours) to 7.69% for the longest exposure time (40 hours; χ^2^ = 23.50, *P* < 0.001). Of the 685 passengers investigated, 349 shared a car with the index case. The RR of attack rate among the passengers who had shared a car with a confirmed case-patient was 3.62 to 27.77 times that of passengers who had been exposed in other cars (χ^2^ = 12.76, *P* < 0.001, Table 2). The average temperature was approximately 23°C at night and 30°C during the day in the 4 provinces through which the train passed. The attack rate in car 12, where the index case travelled, was higher (7.14%) than in car 11, which was air-conditioned (5.13%).

**Table 2.** Influenza attack rate by car number on train 1223, China, 2009

Passenger location (car)	No. of passengers investigated	No. of confirmed cases	Attack rate, % (95% confidence interval)	Relative risk (95% confidence interval)
11	78	4	5.13 (1.41–12.61)	18.11 (2.05–159.68)
12	84	6	7.14 (2.67–14.90)	25.77 (3.15–210.99)
14	187	2	1.07 (0.13–3.81)	3.62 (0.33–39.67)

Others	336	1	0.30 (0.01–1.65)	1.00 (Reference)

χ^2^ = 12.76, *P* = 0.001.

There was a dose-response relationship between time of exposure (time spent in the train) and attack rate, which increased from 0% among the 413 passengers who were exposed for less than 10 hours to 1.13% among the 796 passengers with exposures exceeding 30 hours (Table 3).

**Table 3.** Attack rate according to the time spent on board train 1223, China, 2009

Time traveled (hours)	No. of passengers	No. of cases	Attack rate, % (95% confidence interval)	Relative risk (95% confidence interval)
<10	413	0	0.00	—
10–20	651	2	0.31 (0.04–1.11)	1.00
20–30	315	2	0.63 (0.08–2.27)	2.07 (0.15–28.70)
30–41	796	9	1.13 (0.52–2.14)	3.71 (0.76–35.39)
Total	2175	13	0.60 (0.32–1.02)	—

χ^2^ = 9.10, *P* < 0.05.

Among the 343 contacts exposed to the 13 confirmed first- and second-generation case-patients who had been sick while on the train, 3 (0.87%) developed 2009 ARI (third generation). Among the 40 close contacts of the 3 third-generation case-patients, another 3 developed ARI (fourth generation). Finally, in addition to the 19 confirmed cases with ARI, 3 asymptomatic cases were also identified during medical observation.

## DISCUSSION

Outbreaks of disease have often been reported after air travel. However, outbreaks on trains are less commonly reported. Our investigation in China indicates that train travel played a role in this 2009 H1N1 outbreak. The most plausible explanation for the transmission of 2009 H1N1 virus among passengers and crew members of the train was that the index case-patient in car 12 was infected while on board and transmitted the virus to the crew, who then spread it to other crew and passengers in other cars. Other explanations cannot be ruled out. Indeed, other passengers or crew might have acquired infection before boarding the train and experienced longer incubation periods. All case-patients who were suspected of acquiring infection while on board shared a car. The long distance and duration of the trip (41 hours) as well as close contact in the train may have increased the probability of communicable disease transmission.^18^ We do not know when the index case-patient was infected, but we inferred that she acquired infection before boarding the train, after which she spread it to other passengers and crew.

We identified 13 cases, in cars 11, 12, and 14. Those who shared a car with a case-patient and those who spent more time on the train had higher risks of disease, especially those who sat close to another case-patient. Transmission among passengers was more likely to have occurred among close contacts, such as those who stayed together in the same car. Likewise, long exposure increased the probability of infection.^19^ Passengers who shared a room were in a higher-risk environment for infection.^20^

Crew members, who served many passengers in the train, were at higher risk of infection and, once infected, may have been more effective sources for spreading illness to other crew and passengers in other cars. In our investigation, the longer the time spent on the train, the higher the risk of infection. We attempted to interview all contacts with possible exposure to the confirmed cases, and those contacts were quarantined. In addition to the 13 case-patients who were on the train, the 3 third-generation cases had clear close contact with confirmed second-generation cases, including sharing the same room, providing care for a patient as a nurse, or being a duty waitress in the quarantine hotel. Likewise, 3 fourth-generation case-patients all reported exposures to third-generation case-patients. The attack rate among passengers in our study was similar to that reported for other vehicles.^21^

Among 343 contacts who had been quarantined for 7 days, 3 (0.87%) developed 2009 H1N1, suggesting that management is less effective for diseases with vigorous infection capability and that strict quarantine is necessary,^22^ as was the case for control of severe acute respiratory syndrome (SARS) in 2003.^23^ Although active surveillance in health care facilities played an important role in identifying patients with ARI, the media also assisted in providing important information to the public.^24^ During our investigation, 3 patients contacted the local CDC after developing symptoms. For urgent public health action, use of press releases can educate people, raise awareness, promote health assessment, and accelerate infection containment.^25^

Passengers who travel by air provide detailed contact information. Hence, they can be reached after arrival. In contrast, train passengers could not be reached because tickets are often sold without any identifying information. This could be an issue in cases of serious disease. Potential spread though trains is more difficult to control, as passengers are lost to follow up. This highlights the need to be prepared to respond to any new emerging disease threats.

Until the middle of May 2009, there was only 1 case of 2009 H1N1 infection on a transportation vehicle, which triggered implementation of emergency plans by the passenger ship industry and the responsible authorities. This might constitute overreaction to the event in relatively mild pandemics. However, establishing and maintaining a surveillance system for ARI among train passengers, with the goal of systematic data collection, might help in determining baseline levels of illness. This would enable early identification of outbreaks and timely implementation of control measures, which might prove critical with a disease that has higher illness rates and a greater risk of severe illness.^26^^,^^27^

Based on our observations, the incubation period was 0 to 3 days for influenza A/H1N1. Therefore, we suggest that the appropriate duration of quarantine is 4 days. Longer quarantine would burden the health system and would not be cost-effective. Transportation by train is common in China and other nations. Frequent transport alters the model of disease transmission for new and re-emerging diseases, including 2009 H1N1 and tuberculosis, which can both spread in trains. Because it is more difficult to identify sources of infection, control of communicable diseases may be more challenging in this setting. In our investigation, among 685 persons examined, only 13 cases were confirmed. This low attack rate suggests that active surveillance and management was not a highly effective strategy.

There are several limitations to this investigation. First, we were unable to identify exact chains of person-to-person transmission among case-patients. We could only make inferences on the basis of epidemiologic investigation. Secondly, cases were self-reported and thus subject to reporting errors and underreporting. As a consequence, we were unable to estimate how many people were infected on train 1223. We might have overestimated the attack rate if we did not miss cases and if there were more people in the train than registered. We might have underestimated the attack rate if we missed cases of infection.^28^

Overall, we identified 22 cases of 2009 H1N1 infection, including 19 illnesses, associated with train 1223. Close contact and longer time on board may have contributed to the transmission of pandemic influenza H1N1 virus in the train. As is the case with other major modes of transportation, trains play an important role in transmission of communicable disease.
